# Cognitive Science Honors the Memory of Jeffrey Elman

**DOI:** 10.1162/opmi_e_00023

**Published:** 2019-02-01

**Authors:** Richard N. Aslin, Roger P. Levy

## Abstract

*Jeff Elman (1/22/1948–6/28/2018) was a major and much beloved figure in cognitive science, best known for his work on the TRACE model of speech perception, simple recurrent network models of the temporal dynamics of language processing, and his coauthored monograph*, Rethinking Innateness. *Beyond his individual and collaborative research, he is widely recognized for his lasting contributions to building our scientific community. Here we celebrate his contributions by briefly recounting his life’s work and sharing commentaries and reminiscences from a number of his closest colleagues over the years.*

## RICHARD N. ASLIN (HASKINS LABS) AND ROGER P. LEVY (MIT)

Jeffrey Locke Elman dedicated his career to the study of human language processing and learning, investigating how humans use language flexibly and productively, and how these abilities can be learned from linguistic input. He obtained his PhD in Linguistics at the University of Texas at Austin in 1977 and immediately joined the University of California, San Diego, where he spent the rest of his career. Jeff started as a faculty member in the Department of Linguistics, and his earliest work focused on phonetics and phonology. These seminal studies presaged the core ideas for which he is now best known: seemingly discrete combinatorial units of language, such as phonemes, may be best understood as emergent properties of underlying continuous multidimensional representations, such as phonetic input.

The late 1970s at UC San Diego was a time of tremendous intellectual ferment in cognitive science, and Jeff quickly joined what became known as the connectionist movement, which used artificial neural networks—computational models inspired by biological neural networks—to construct theories of distributed representation and parallel processing in cognition. Jeff’s colleagues in this movement included David Rumelhart, Jay McClelland (with whom he codeveloped the TRACE model of speech perception), Liz Bates, Geoffrey Hinton, Marta Kutas, Paul Smolensky, and Terrence Sejnowski. The connectionist movement, offering both novel theoretical ideas and powerful learning models for engineering, had a transformative effect on cognitive science. Jeff’s work on Simple Recurrent Network models, introduced by his 1990 article “Finding Structure in Time,” proposed that time-evolving continuous hidden-state representations are fundamental to language processing, enabling prediction-based unsupervised learning of language structure. This work remains among the most influential in the history of cognitive science.

Jeff played a major role in advancing cognitive science as a field of study. At UC San Diego, in 1985 he and colleagues cofounded the interdisciplinary Center for Research in Language, where he was the Founding Director, and in 1986 the first Cognitive Science department in the world, which he chaired from 1995 to 1998. Jeff served in many other administrative capacities during his career, including Dean of Social Sciences and cofounder of the Kavli Institute for Mind and Brain and the Halicioğlu Data Sciences Institute. He also contributed through professional service outside his home university, including as President of the Cognitive Science Society and Chair of the National Institutes of Health study section on Language and Communication.

Throughout his administrative service, Jeff maintained an active research program. In the later stages of his career, he focused on event knowledge in language processing using computational, behavioral, and neurolinguistic methods. His intellectual legacy includes not only his research output but also the many students and collaborators who have learned from and revere him. But even more impressive than his scholarly accomplishments, Jeff was unfailingly generous to and supportive of everyone in his field, from entry-level students to his most senior colleagues. He was rigorous in defending his perspective on cognitive science but always respectful of alternative viewpoints. He promoted his methodological and theoretical preferences, including hosting many researchers (including one of us [RNA] for an unusually rainy month in La Jolla in 1990), yet he was always willing to listen to differing opinions. Colleagues like Jeff Elman are rare indeed, and he will be sorely missed.

## MARTA KUTAS (UCSD)

*I miss my morning coffee ritual*. Jeff and I routinely met for coffee early in the morning on the UCSD campus. We never sat down, but between the walk to and from the Cognitive Science building we talked business and pleasure. It was a rare day that Jeff and I did not meet (and greet with extended interaction) at least one person whom he knew; his world of friends and acquaintances was remarkably extensive.

Of our many discussions, one of particular pertinence here was, what is cognitive science? Jeff’s contribution to cognitive science is legendary and yet he too found defining cognitive science a “hard question” with “no single answer.” “It’s one we should always ask but we should be wary of definitions that are limiting.” Of course, this “does not mean anything goes” but it “does mean we should be open to being stretched”—that is, keep an open mind. Jeff believed that “a real key will be not to define any given subject matter *X* per se as being in or out of cognitive science. It’s a matter of how *X* is studied and the broader views one has about *X*. Two different people can study the same thing, and one might be better off in CogSci and the other in another department.” Jeff grappled with a lot when he moved from Linguistics to CogSci. More than once Jeff said, “I wasn’t sure I belonged, but now very much feel I do!” Indeed, he did!

When the department was formed, it grew out of intense interactions that gave rise to new ideas and frameworks such as distributed cognition, neural networks, and cognitive linguistics, among many others. People realized these interdisciplinary discussions were resulting in groundbreaking new possibilities in areas that they cared about deeply. And, although there has not been an explosion of cognitive science departments, Jeff felt that the “impact of cognitive science thinking has been huge. Concepts, frameworks, and methodologies in many different fields are different because of cognitive science as a field,” such as cognitive neuroscience and machine learning. “The whole vocabulary that many of the established disciplines use has been changed because of insights from CogSci.” Many of those insights are Jeff’s legacy.

## JAY MCCLELLAND (STANFORD)

I had the privilege to work closely with Jeff Elman for a few years in the early 1980s, and I followed his subsequent work closely through his seminal paper “Finding Structure in Time” and his related work from the early 1990s. What I appreciated the most about Jeff was his ability to question the most deeply held tenets of linguistics, and to offer alternatives to conventional wisdom, through both behavioral experiments and computational modeling.

In our work on the TRACE model of speech perception, Jeff came up with a critical experimental prediction testing the central claim of the model, a claim that was considered logically incoherent at the time and remains controversial to this day: that word-level information can feed back into the apparatus of speech processing to determine the actual *sounds* someone hears. The phenomenon Jeff predicted, and that we experimentally confirmed, is that lexical knowledge could penetrate the mechanism of speech perception to a deep enough level that it could produce knock-on effects on the perception of other speech sounds—even speech sounds in subsequent words. The findings were very provocative and led to a series of further studies attempting to refute or support them—an outcome that indicates just how important the experiment was.

During the time I worked with Jeff, a major revolution began to occur in our conception of language representation and learning. When we started out, there were phonemes and there were words and it seemed to make sense to propose that these conceptual units actually corresponded to representational primitives in the mind. As connectionist modelers discovered distributed representations, though, they began to question these units, and Jeff was the first to realize how profoundly transformative a more distributed conception of these units might be. I remember an impassioned presentation in which Jeff described a distributed neural network that could repeat, in Jeff’s own voice, “This is the voice of the neural network.”

Jeff went on from there to his series of important papers beginning with “Finding Structure in Time.” He showed that simple recurrent networks could learn distributed representations that captured key aspects of linguistic categories like noun and verb and key regularities of language such as long-distance dependencies without explicitly representing such categories or the rules by which they could be combined to form sentences. Today, a huge body of language research continues to explore models applying and extending these ideas.

## GARY COTTRELL (UCSD)

I knew Jeff for at least 33 years, since I moved to San Diego in 1985 and began attending PDP group meetings. I was there in the PDP group meeting when he first presented his simple recurrent networks. I was surprised it worked, and I didn’t believe it at first (and said so!)—how could it remember further back in time by only learning from the previous time step? Obviously, I was wrong. It was really exciting to see this, and I immediately started playing with them. Not many people have network architectures named after them by the community.

Jeff’s incredible skill was to boil down an issue to as simple a demonstration as possible, and that’s why he called these simple recurrent networks. His demonstrations of their power in “Finding Structure in Time” was a classic example of this. He showed how these very simple models could learn about language by essentially “listening to the radio”—he demonstrated how they could learn both syntax and semantics (albeit ungrounded semantics) simply by the process of trying to predict the next word. It was a tour de force, and it’s no wonder it’s been cited well over 9,500 times.

His career is well-known and appreciated, but Jeff the person always exuded a kind of eagerness, excitement, and enthusiasm for everything he was interested in. For example, he became a docent at Torrey Pines State Park, which took him three months of training. Talking about it with him, he made ME want to be a docent at Torrey Pines. He expressed how he now saw the world differently because of his deeper knowledge of plants and animals.

The other part of Jeff that I loved was how he always gave me good political advice when negotiating issues with the campus. Unfortunately, I’m not diplomatic enough to follow his advice, but it was always useful.

I will miss Jeff terribly, but his spirit will live on at UCSD in the many people whose lives he touched here and around the world. Rest in peace, Jeff. You deserve a rest after all that you have done to change the world.

## CAROL PADDEN (UCSD)

I first met Jeff in 1978 when I came to UC San Diego as a graduate student. He was my phonetics professor my first quarter. Thirty years later, I became his Associate Dean when he was Dean of Social Sciences. He caught me by surprise when he made the offer. I had never thought of myself in that capacity before. But that’s how Jeff did everything in his career: he was an unconventional scholar, an unconventional colleague, and an unconventional Dean.

He broke every rule of what an “effective leader” should do. He shared his time freely because he saw time not as something to hoard or to jealously guard, but to give whenever possible. As generous as he was, he was always effective and efficient. He was the first to apologize if something was done wrong or insensitively. He was compassionate to his colleagues, but he could tell the truth. Above all, the gift he had as Dean was that he liked the puzzle of institutions, and how to make them work in favor of human beings.

He liked building programs, departments, institutes, initiatives, centers—and he did! He saw a moral imperative to make ideas possible. He liked the exercise of bringing together people to see what new ideas they might create. His legacy is here for everyone to see: the first Department of Cognitive Science in the world at UC San Diego and, just last year, a new Data Science Institute that he cofounded just before he passed away. All of what he built, he built durably, so others like myself could step in and pick up from where he left off.

It was a shock to lose him. He saw a university campus not as a lumbering, slow-moving beast of people and buildings, but as a force of possibility on a very large scale. He loved good ideas, and he loved innovative good ideas even better. Those of us who worked closely with him know very acutely what we lost in a heart-breaking moment on a summer day in June: a dear friend and an extraordinary human being.

## MARK JOHNSON (UNIVERSITY OF CAMBRIDGE)

As one of the coauthors of *Rethinking Innateness* (MIT Press, 1996) led by Elman, I had the privilege of seeing Jeff’s masterful academic and personal leadership skills close up. Following on from the highly successful connectionist summer school for developmental psychologists that Jeff ran at UCSD until 1991, he assembled a talented but challenging group of coauthors with different perspectives on key issues. Jeff successfully prompted, cajoled, mediated, soothed, and distilled until our fiery debates became coherent and cohesive text. There is no doubt that the great success of the book was largely due to him.

After the publication of the book, Jeff became a regular visitor to London, in later years joined by his spouse Ray Eller. London became a second home, with a network of UK friends and colleagues devastated by news of his death. In fall 2017, Jeff (and Ray) provided comfort and support during the terminal illness of my late wife, Annette. It was typical of Jeff that he rescheduled his busy diary in order to visit London during Annette’s final months, something for which I will be eternally grateful.

We have lost not only an exceptional scientist and leader, but also a loyal and empathic friend.

## JANET WILES (UNIVERSITY OF QUEENSLAND)

Nearly 30 years ago a young postdoc from Australia wrote to Professor Jeff Elman at UCSD, asking technical questions about replicating his simple recurrent network (SRN) simulations. “Finding Structure in Time” was a tour de force, showing new ways to think about linguistic structure, and by extension, also cognitive and computational structure. This network learned parsimonious representations of temporal structure. Elegant!

But what kind of computational machine was this? I spent my first sabbatical with Jeff arguing about this question. Jeff was a linguist first and foremost, but also adept in many different fields. He was inspiring to work with, as he asked fundamental questions, and applied intuition and rigor in equal measure to explore them. Jeff loved dimension reduction tools for investigating SRN dynamics, and by visualizing the hidden unit spaces, we could see the trajectories oscillate as sequences were processed. Jeff recruited Paul Rodriguez to our joint project, who mathematically explored how the hidden layer created multiple oscillators with linked dynamics, oscillating toward a fixed point to count the first part of the sequence, then remapping the network dynamics to oscillate away from an unstable saddle point for the second part of the sequence. The SRN learned convergence and divergence rates that were exact inverses of each other. Through this and many other studies the SRN started a new way of thinking about the representation and processing of temporal structure across the cognitive sciences. It was a privilege to be part of one of those studies.

Jeff had a flair for starting things. Not only did he write back (within 24 hours!) to that young postdoc, and host her first sabbatical, he became a long-term mentor, friend, and fellow traveler into many intellectual realms. Through the 1990s he provided advice on multidisciplinary collaboration for my university’s Cognitive Science program. In the early 2000s, time-encodings turned up again in a very different research context—the functional role of new brain cells in adults. Jeff introduced me to Rusty Gage and Brad Aimone at the Salk Institute, who were developing a computational model of new-born granule cells in dentate gyrus. He brokered and took an active interest in our early meetings, and the Kavli Institute for Brain and Mind supported the model’s early development and later experimental work with Andrea Chiba’s lab over the following decade. In subsequent years, Jeff served on the board of the Australian Research Council’s Thinking Systems Initiative (2006–2011), and most recently on the advisory board of the ARC Centre of Excellence for the Dynamics of Language (2014–2018). Fun to work with, intellectually challenging, Jeff would listen wholeheartedly and was always worth listening to.

Jeff—For your intellectual excitement, wise council, and friendship, *thank you*. You are sorely missed.

## ELISSA NEWPORT (GEORGETOWN)

There are so many things I remember and miss about Jeff. But perhaps the most important thing is that he was the nicest person I have ever known.

I met Jeff in the Xerox room at UCSD during his first year as an Assistant Professor, but we didn’t get a chance to know each other very well at that time. My interactions with him were primarily later, when our field had become more divided and sometimes quite argumentative. Jeff and I were on different sides of the field, but we understood that we agreed with each other on many important issues. I served on the Language and Communication review panel at NIH when he was its chair, and he set a generous, warm tone to our discussions; thanks to this spirit, we always discussed research from all fields with respect and supported funding the best of breed from all perspectives; we never advocated just for research from our own points of view. That’s what Jeff always exemplified to me.

In our own work, Jeff and I both wrote about the advantages of childhood (his “Starting Small” and my “Less Is More”). While some (at first even me) might have viewed these papers as competitive (who said it first?), Jeff invited me to write a paper with him and I accepted with great pleasure. We never did it, but it meant a lot to me that he asked me.

The day after he learned about the Rochester case of sexual harassment and my involvement in going after someone who harassed students and faculty, he called me and we talked for two hours. He thanked us for our actions, he wanted to know what he could do to help. We dreamt up tens of things we could do to stamp out sexual harassment in our field.

My last few conversations with him were after his heart attack. As soon as I heard, I called Ray to find out what had happened and give him my warmest wishes for Jeff; I didn’t want to bother Jeff because I knew he was so sick. But Jeff soon called me back. He wanted to thank me for thinking of Ray. That was so incredibly kind and thoughtful of him—thoughtful to Ray, thoughtful to me.

I thought and hoped he would be fine, I never imagined we would lose him. I dearly miss having someone so generous, warm, and kind in my life. Our field must remember him for the example of kindness and generosity he represented for all of us.

## KEN MCRAE (UNIVERSITY OF WESTERN ONTARIO)

I had the extraordinary pleasure of collaborating with Jeff for 20 years. That’s a long time! I loved working with Jeff. I learned so much from him. I continue to try to emulate Jeff in a number of ways. An important way is that I try to ask myself what Jeff consistently asked whenever we were generating ideas: “What’s most interesting about this?”

As is the case with great scientists, Jeff had a lot of fabulous, innovative ideas, he was fearless in terms of getting them out there, and he always saw the big picture. Jeff was an awesome writer. His ability to communicate ideas and to couch things at a high, centrally important theoretical level was second to none. In fact, because of his ability to take a study that Mary Hare and I, as mere mortals, saw as pretty neat, and turn it into a study with wildly important, theoretically broad implications, we used to call him the “Fluffmeister,” which I’m pretty sure he didn’t terribly appreciate.

But the best part of working with Jeff for the past 20 years has nothing to do with science. Many of us love and miss Jeff so much because, in addition to being an extraordinary scientist, he was an even better person. He was incredibly warm, kind, patient, and generous with his time. I witnessed many times when he was quick with lavish praise, especially with young people. Jeff was most definitely a passionate fighter for equity and social justice during his entire adult life, and the importance of this cannot be overstated. He deeply loved Ray, his children, their spouses, and his grandchildren (Emily, Nate, Stella, & Oliver; Jeremy, Erin, Henry, & Elise).

At the 2018 Cognitive Science conference a month after Jeff died, Jay McClelland said to me, “You’re so lucky. I got to work with Jeff really closely for only a few years. You got 20.” And Jay is right. I’m so happy that I got to collaborate with Jeff, and more importantly, got to have him as a dear friend.

## FRED DICK (BIRKBECK/UCL)

I had the unbelievable luck of doing a PhD in Cognitive Science at UCSD with Liz Bates and Jeff starting in the late 1990s. My first lab rotation was 3 months of working through the just-published *Rethinking Innateness* connectionist exercises in tlearn every week with the author himself. Not only was this an introduction to Jeff’s genuine kindness and (usually) superhuman patience—he had to show me how to cd ../ more than once—but to his unique ability to make complex or abstract ideas utterly transparent. I think this was partly an outcome of his real passion for understanding process and mechanism, and not only when he was working on modeling language. During Jeff’s last visit to London, we went to an exhibit on the Russian revolution at the British Library. I remember feeling I had learned a lot more from his thoughts on the potential “tipping points” in this period of history than from the exhibition itself.

Liz said more than once that every article Jeff wrote was a jewel. I felt the same way about any time we spent together. Regardless of how busy he was, Jeff always gave you his full attention and focus, and whatever ideas you might have come in with came out a lot better thanks to him.

Writing just a few sentences about Jeff in the past tense has involved a lot of staring at a blank screen. A thousand times more words could not do justice to his generosity, warmth, integrity, creativity, and intellect. I miss him immensely.

## SUSAN GOLDIN-MEADOW (UNIVERSITY OF CHICAGO)

If I had to choose one word to describe Jeff, it would be *generous*. Jeff was generous with his formidable intellect—he was willing to think with you about anything and he did it so well. He was generous with his time—he made time for everyone and had a way of using Harry Potter–like skills to expand what, for everyone else, was a limited resource. He was generous with his friendship—he was liked, valued, and indeed cherished by everyone who was lucky enough to call him a friend. And he was a deeply thoughtful friend. My favorite example is Sam. Jeff and I were both speakers at an AMLaP conference in Lancaster, England. The hotel where we stayed placed a charming stuffed dog, named Sam, in every room—you could purchase the dog and contribute to a charity. I bought two, one for my daughter and one for my grandchildren. Jeff bought one for his grandchildren. When we got home, Jeff confessed that he couldn’t part with Sam and kept him for himself. I did give mine away but said I should have bought three. A week later, a package arrived from England and I knew, without opening it, that Jeff had contacted the hotel and sent me a Sam of my own. Jeff did things, big and small, that made the world a better place, and Sam is now my daily reminder of how important that is and how very special he was.

## JAMIE ALEXANDRE (LEARNING EQUALITY, INC.)

Our hearts break, because the world still had so much to learn from you. But like the recurrent neural networks you created, we’ll take everything you taught us and feed it back in, again and again, changing hearts and minds, cascading and echoing across time and through history. As we glide through the state space of our lives, you’ve been there tweaking the weights to guide us toward an ever-better world. You wove a tapestry of minds that strive toward a common goal: a more vibrant world, a more just world, a world where everyone can flourish and learn. This will be your legacy: the hearts you touched, the minds you changed, the seeds you planted.

As a student of yours, I learned what true mentorship looks like. You were there when I needed you—and when I didn’t, you stepped out of the way. You let me try things and fail, because you knew the wisdom that comes from walking to a dead-end is far greater than being told not to try. You had my back when others tried to push me off course. You taught me that life can’t all be work, but that work can be joyful too. You tended to me like a garden, or a neural network being trained: making sure the conditions were right, and I had what I needed, then letting me learn and grow on my own.

With deep love and gratitude, on behalf of the countless students you helped over the years.

I want to close by quoting Jeff himself: “That’s probably the biggest gift you can give people: to take your passion and *share* it, and have things beat in the hearts of others that beat in yours.”

## ARIELLE BOROVSKY (PURDUE)

Jeff became my mentor by accident. I had enrolled in a single-semester research rotation with him in my first semester of graduate school, while my official mentor, Liz Bates, was on sabbatical. Liz, sadly, only lived a year after that sabbatical, and Jeff kindly took me under his wing. So the rotation turned into a master’s thesis and later evolved into a doctoral dissertation, and then, a postdoc. And, he continued to advise me after I *finally* launched into a faculty position. His mentorship lasted for more than 15 years.

Jeff was an extraordinarily generous person, and this generosity extended to his mentees. He shared so much during our meetings. Much of what you would expect: insights on current research, his advice about my academic progress, or lack of it. Jeff also loved to gossip—with a generally positive tone. He would beam about his friends, far and wide, who did extraordinary things. He shared his own life—his joy at becoming a grandparent, the challenges of his father’s illness, his love of vacations and sailing with Ray, and his budding interest in hiking and cataloging the flora of Torrey Pines. He was unfailingly positive. I can recall only a handful of times where he expressed true irritation. Once when he was angered by national politics, and another, in response to a poor rendition of Queen’s “Bohemian Rhapsody.”

My favorite time with Jeff was the year he took sabbatical while I was a postdoc. Jeff had often lamented how he wanted more time to focus on research, and both of us found ourselves at a point where we could focus entirely on research. I think happily about days where we would almost skip between his office and the lab to test out recently hatched ideas.

I recognize now that even in his informal interactions, Jeff was carefully cultivating a diverse and rich professional training set, helping me tweak the parameters of my network to avoid entrenchment and local minima. And occasionally providing a helpful push. His mentorship had an immense impact in ways that are hard to fully fathom. What I can say, without qualification, is that my life and my science are better because of him. And, while I wish I could thank him directly, his influence continues to live on as I so often hear his words echoed when I now mentor my own students.

Jeff made an enormous contribution to our field on the value of learning structure in time. Jeff himself was deeply learned, with great structure of character. I just wish there had also been more time.

